# A Kinetic Pump Integrated Microfluidic Plate (KIM-Plate) with High Usability for Cell Culture-Based Multiorgan Microphysiological Systems

**DOI:** 10.3390/mi12091007

**Published:** 2021-08-24

**Authors:** Kenta Shinha, Wataru Nihei, Hiroko Nakamura, Tomomi Goto, Takumi Kawanishi, Naoki Ishida, Nao Yamazaki, Yuki Imakura, Shinji Mima, Kosuke Inamura, Hiroshi Arakawa, Masaki Nishikawa, Yukio Kato, Yasuyuki Sakai, Hiroshi Kimura

**Affiliations:** 1 Department of Mechanical Engineering, School of Engineering, Tokai University, 4-1-1 Kitakaname, Hiratsuka 259-1292, Japan; shinba04@gmail.com (K.S.); nihei@dpc.agu.ac.jp (W.N.); hironaka@tokai-u.jp (H.N.); tomomi557.0512@gmail.com (T.G.); 2 Micro/Nano Technology Center, Tokai University, 4-1-1 Kitakaname, Hiratsuka 259-1292, Japan; 3 Faculty of Pharmacy, Institute of Medical, Pharmaceutical and Health Sciences, Kanazawa University, Kanazawa 920-1192, Japan; tk723@stu.kanazawa-u.ac.jp (T.K.); ishida0117@stu.kanazawa-u.ac.jp (N.I.); arakawa@p.kanazawa-u.ac.jp (H.A.); ykato@p.kanazawa-u.ac.jp (Y.K.); 4 Bio Science & Engineering Laboratory, FUJIFILM Corporation, 577 Ushijima, Kaisei-machi 258-8577, Japan; nao.a.yamazaki@fujifilm.com (N.Y.); yuki.imakura@fujifilm.com (Y.I.); shinji.mima@fujifilm.com (S.M.); 5 Department of Chemical System Engineering, Graduate School of Engineering, The University of Tokyo, 7-3-1 Hongo, Tokyo 113-8654, Japan; k_inamura@chemsys.t.u-tokyo.ac.jp (K.I.); masaki@chemsys.t.u-tokyo.ac.jp (M.N.); sakaiyasu@chemsys.t.u-tokyo.ac.jp (Y.S.)

**Keywords:** microphysiological system (MPS), organ-on-a-chip, kinetic pump, coculture, cell-based assay

## Abstract

Microphysiological systems (MPSs), including organ-on-a-chip (OoC), have attracted attention as a novel method for estimating the effects and side effects of drugs in drug discovery. To reproduce the dynamic in vivo environment, previous MPSs were connected to pump systems to perfuse culture medium. Therefore, most MPSs are not user-friendly and have poor throughput. We aimed to develop a kinetic pump integrated microfluidic plate (KIM-Plate) by applying the stirrer-based micropump to an open access culture plate to improve the usability of MPSs. The KIM-Plate integrates six multiorgan MPS (MO-MPS) units and meets the ANSI/SBS microplate standards. We evaluated the perfusion function of the kinetic pump and found that the KIM-Plate had sufficient agitation effect. Coculture experiments with PXB cells and hiPS intestinal cells showed that the TEER of hiPS intestinal cells and gene expression levels related to the metabolism of PXB cells were increased. Hence, the KIM-Plate is an innovative tool for the easy coculture of highly conditioned cells that is expected to facilitate cell-based assays in the fields of drug discovery and biology because of its usability and high throughput nature.

## 1. Introduction

Microphysiological systems (MPSs), including organ-on-a-chip (OoC), which can reproduce the in vivo blood flow and organ/tissue structure by culturing cells on a microfabrication technology-based chip, are attracting attention as a technology that will revolutionize drug research and development [1,2,3,4]. MPSs are expected to become powerful tools for predicting pharmacokinetics in various organs and tissues, such as absorption, distribution, metabolism, and excretion (ADME) and drug effects including toxicity. In addition to single-organ models [5,6,7,8,9,10,11], multiorgan MPSs (MO-MPSs) have been proposed to evaluate interorgan interactions by coculturing cells derived from multiple organs and tissues [12,13,14,15,16]. The greatest advantage of MO-MPSs is the ability to evaluate interorgan interactions in real time. MO-MPSs may contribute to improving the accuracy of the physiologically based pharmacokinetic-pharmacodynamic (PBPK-PD) model used to predict pharmacokinetics, because they can evaluate drug effects according to drug concentrations that change with time [17,18]. Some MPSs are commercially available from several companies, such as Emulate, TissUse, and Mimetas [19]. A market report by Yole estimated the MPS market revenue to be less than USD 7.5 million in 2016; however, the MPS market has the potential to grow tremendously in the medium to long term and become a multibillion-dollar market due to its potential to help save billions of dollars annually by bridging the gap between preclinical and clinical trials required for drug development [20].

A system for pumping the culture medium is essential for reproducing the blood flow in MPSs. Using a syringe pump or a peristaltic pump is the simplest pumping method [21,22,23,24]. The flow rate and pumping type (perfusion or one-through) can be selected depending on the selected pump because the MPS and pump are connected via tubing. However, connecting the pump to the MPS is a very complicated process due to the need to avoid contamination and the inflow of air bubbles. In addition, an increased volume of culture medium is required to be kept in the syringe or reservoir, which increases the cost of the experiment and reduces the reactivity. An MPS integrated with a perfusion system mimicking a peristaltic pump has been developed as an alternative to external pumps [25,26]. In this method, all or parts of the channel structures of the MPS are fabricated from a highly elastic material, and the channels are closed and opened by applying external pressure. The flow can be formed by performing these processes continuously at several points in the flow channel. The dead volume of the culture liquid, which was one of the problems of the external pump system, was reduced by integrating the pump with the MPS. However, the operation remains complicated because it is necessary to connect a tube to load air pressure to pressurize the MPS. Furthermore, a pneumatic pressure-driven MPS, which uses air pressure at the top of the culture chamber to pump liquid based on the pressure difference, has been proposed [27,28]. This MPS reduces the complexity of experiments because eight units can be perfused in a single system. However, the liquid level in the culture chamber is changed because the pressure is applied directly to the culture chambers and the solution is pumped back and forth between the chambers. The difference in the height of the cell culture insert used for culturing membrane-type organ cells between the apical (AP) and basolateral (BL) sides causes potential flow, which may interfere with the barrier function. Therefore, the function of cell transporters cannot be accurately assessed under perfusion culture. A potential flow-based pumping system is used in many MO-MPSs as it does not require an external pump [16,17,18,29,30,31]. The MO-MPS using potential flow has two or more chambers. The flow is controlled using potential energy by tilting the MO-MPS to generate a difference in the liquid level in the chambers. Wang and Shuler achieved unidirectional perfusion by optimizing the channel design, enabling the long-term culture of cells under the shear stress condition [32]. In this method, the flow is generated according to the change in the height of the liquid surface, and the cell culture operation is complicated because it is necessary to perform cell culture in a closed channel or in a culture section that is compartmentalized from the pumping channel by a porous membrane. In addition, it is necessary to perform preculture and evaluation in the MO-MPS because the cells are seeded onto the MO-MPS directly. This cumbersomeness may reduce usability and throughput.

We previously developed a stirrer-based kinetic pump as an on-chip pumping system. The kinetic pump can perfuse the flow channel by rotating the stirrer bar installed in the pump chamber in the flow channel [33,34]. Previous studies have shown that MPSs with kinetic pumps are useful for drug assay tests [35,36]. However, as our proposed on-chip pump MPS is a closed channel system, it is difficult to use to culture primary and induced pluripotent stem cell (iPS)-derived cells, which require differentiation induction after seeding, and to culture cells in a hierarchical manner that more closely resembles the tissue structure, because seeding and media exchange are complicated. To realize a higher quality assay system, it is necessary to maintain organ or tissue models that mimic the in vivo biological functions within the MPSs. In addition, to utilize the MPS in the drug discovery field, the throughput of experiments must be increased.

In this study, we have developed a kinetic pump integrated microfluidic plate (KIM-Plate) by applying the stirrer-based kinetic pump to an open access culture plate to improve the usability of MO-MPS. The KIM-Plate integrates six MO-MPS units and meets the American National Standards Institute/Society for Biomolecular Screening (ANSI/SBS) microplate standards (width 128 mm × depth 85 mm × height 40 mm). The KIM-Plate can be used in the same way as conventional cultureware, because it realizes perfusion culture between two wells of the same size as those in a 24-well plate. Evaluation of the mixing ability of the kinetic pump and culture experiments of primary hepatocytes derived from chimeric mice with humanized liver tissues (PXB cells) and human induced pluripotent stem cell derived small intestinal epithelial cells (hiPS intestinal cells) were conducted for evaluation of the KIM-Plate. Based on the results of transportation evaluation, it was confirmed that the stirrer-based kinetic pump had a sufficient pumping capacity. Coculture experiments of intestinal cells and hepatocytes showed the effectiveness of the plate for coculture experiments, indicating that the KIM-Plate is an innovative tool for the easy coculture of highly maintained cells.

## 2. Materials and Methods

### 2.1. Kinetic Pump Integrated Microfluidic Plate (KIM-Plate)

**Figure 1:**
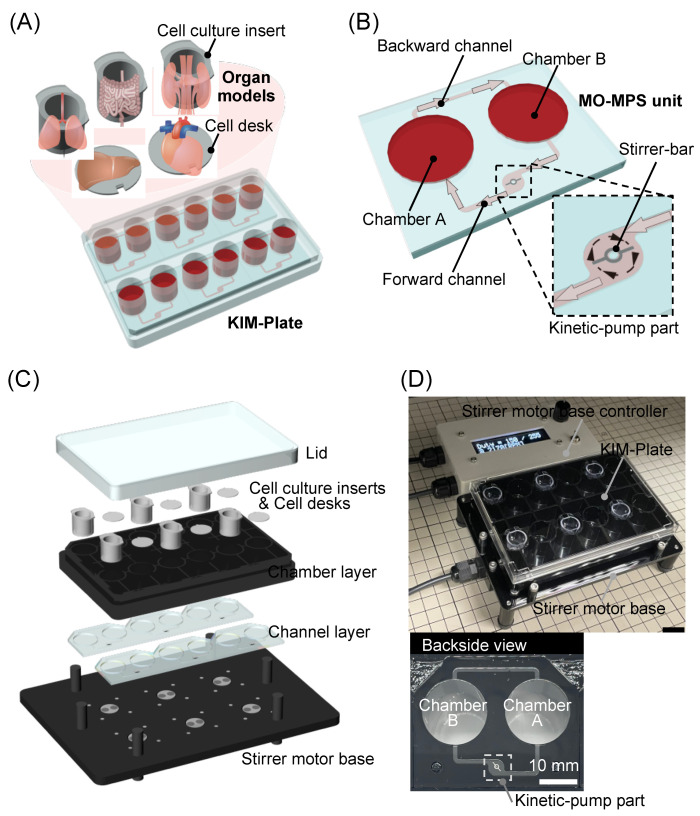
Kinetic pump integrated microfluidic plate (KIM-Plate) for multiorgan cell culture. (**A**) Conceptual diagram of the KIM-Plate. The KIM-Plate has a simple structure mimicking a 24-well plate, allowing easy handling. (**B**) The multiorgan MPS (MO-MPS) unit consists of two culture chambers and microfluidic channels. The stirrer-based kinetic pump is integrated in a forward channel for medium perfusion. (**C**) The KIM-Plate consists of a lid, a chamber layer, and a channel layer and is set on a stirrer motor base to control the kinetic pump and perfuse culture medium. (**D**) Photograph of the KIM-Plate and stirrer motor base system. The KIM-Plate was fabricated by laser fusion of the polystyrene channel and chamber layers. Motor rotation of the stirrer motor base can be controlled by a controller.

We developed a stirrer-based kinetic pump integrated microfluidic plate (KIM-Plate) for multiorgan cell culture as a novel MO-MPS (Figure 1A). The KIM-Plate consists of six independent MO-MPS units and meets the ANSI/SBS microplate standards (128 mm width × 85 mm depth × 40 mm height). The culture chambers of the KIM-Plate are open and have the same shape and size as those in a standard 24-well plate. Thus, the KIM-Plate can be operated in the same way as a conventional culture plate. Furthermore, commercially available cultureware, such as cell culture inserts and cell desks, can be used in the KIM-Plate. The MO-MPS unit consists of the two culture chambers connected by microfluidic channels (1.0 mm width × 0.3 mm height) which are backward and forward flow channels with a kinetic pump part (Figure 1B). In order to reduce the shear stress on cells and to evaluate only the effect of coculture (i.e., chemical interactions such as the paracrine effects), channels were placed 1.2 mm higher than the bottom of the culture chamber. One to four organ models can be cultured in the MO-MPS unit using cell culture inserts and cell desks. The stirrer-based kinetic pump developed in our previous study can perfuse the culture medium by rotating the stirrer bar (3 mm long, 0.2 mm wide) using a stirrer motor under the KIM-Plate [33]. The culture medium flows from chamber B to chamber A by the clockwise rotation of the kinetic pump integrated in the forward flow channel. The same amount of solution as that pumped out flows from chamber A to chamber B in the backward flow channel, and then the solution circulates. 

The KIM-Plate, which was manufactured by Sumitomo Bakelite Co., Ltd., based on our design, consists of two layers, the lower layer of the microchannel structure and the upper layer of the cell culture chambers, and a lid (Figure 1C). In particular, the KIM-Plate was fabricated by laser fusion of the chamber and channel layers of polystyrene (PS) made by injection molding. The KIM-Plate is composed of PS layers and a special stainless-steel stirrer bar, and the adsorption performance is comparable to that of conventional culture plates (Figure S1).

The KIM-Plate is set onto the stirrer motor base to run the kinetic pumps. The stirrer motor base with six magnetic rotors was developed to simultaneously control six kinetic pumps on the KIM-Plate. A DC Tubeaxial Fan (108-AFB0412HHA-TA5F, Delta Electronics Inc., Taipei, China) was used as a motor to rotate the magnetic rotors. The six DC fans are connected to a stirrer motor base controller, and the rotation speed of the magnetic rotors on the fan can be controlled by controlling the current with the controller. The body of the stirrer motor base is made of polycarbonate. The magnetic rotors are made of iron around the magnets to prevent them from having an adverse effect on the electronic base. The base and the controller were manufactured by Microfluidic System Works Inc. based on our design.

### 2.2. Chemical Transportation Characterization of the KIM-Plate

The kinetic pump was driven from 0 to 6500 rpm to study the transportation of red dye and evaluate the circulatory function of the kinetic pump. For the transportation experiment, a red dye solution was introduced into chamber A and ultrapure water was introduced into chamber B of the KIM-Plate, the kinetic pump was driven at 6500 rpm for 120 min, and the transportation of the red dye was evaluated. For the control experiment, the red dye transportation was examined at a stirrer rotation speed of 0 rpm. Furthermore, the transportation of Lucifer yellow CH Dipotassium Salt (LY, 12806271, FUJIFILM Wako Pure Chemical Co., Osaka, Japan) was quantitatively evaluated. For this experiment, 10 µM of LY solution was introduced into chamber A and ultrapure water was introduced into chamber B of the KIM-Plate. The fluorescence intensities of the samples collected at 0 to 120 min were measured using a microplate reader (SH-9000, CORONA Electric Co., Ltd., Hitachinaka, Japan), and the ratio of the fluorescence intensity of chamber B to that of chamber A was determined.

### 2.3. Cell Preparation

A cell desk (MS-0113K, Sumitomo Bakelite Co., Ltd., Tokyo, Japan) and culture insert (353495, Falcon, New York, NY, USA) were used as cell culture substrates in the KIM-Plate. PXB cells (PhoenixBio Co., Ltd., Higashihiroshima, Japan) and hiPS intestinal cells (provided by FUJIFILM Co., Ltd., Tokyo, Japan) were seeded on the cell desk and culture insert, respectively, and differentiated and precultured in conventional 24-well plates. 

PXB cells were cultured at 37 °C in an incubator in a humidified atmosphere containing 5% CO_2_. Dulbecco’s modified Eagle medium (DMEM, D2902-10X1L, Sigma-Aldrich Co., St. Louis, MO, USA) supplemented with 10% fetal bovine serum (FBS, 04-007-1A, Cosmo Bio Co., Ltd., Tokyo, Japan), 44 mM NaHCO_3_ (19101305, FUJIFILM Wako Pure Chemical Co., Osaka, Japan), 20 mM N-2-hydroxyethylpiperazine-N’-2-ethane sulfonic acid (HEPES, 15630080, Invitrogen, Waltham, MA, USA), 1% penicillin-streptomycin (15140148, Life Technologies Co., Carlsbad, CA, USA), 15 µg/mL L-proline (59310032, FUJIFILM Wako Pure Chemical Co., Osaka, Japan), 0.25 μg/mL insulin (I1882-100MG, Sigma-Aldrich Co., St. Louis, MO, USA), 5 ng/mL epidermal growth factor (EGF, E9644-.2MG, Sigma-Aldrich Co., St. Louis, MO, USA), 50 nM dexamethasone (D8893-1MG, Sigma-Aldrich Co., St. Louis, MO, USA), 0.1 mM L-ascorbic acid 2-phosphate (01312061, FUJIFILM Wako Pure Chemical Co., Osaka, Japan), and 2% dimethyl sulfoxide (D2650-5X5ML, Sigma-Aldrich Co., St. Louis, MO, USA) was used as the preculture medium. The preculture medium was changed every 3 days during the 6 days of the preculture.

The preparation of the hiPS intestinal cells was performed according to a previously described method [37]. Briefly, mTeSR1 medium was used to maintain hiPS cells. To differentiate hiPS cells into endodermal cells, they were incubated in serum-free medium containing activin A and then cultured in serum-free medium containing activin A, bone morphogenic protein 4 (BMP4), vascular endothelial growth factor (VEGF), and fibroblast growth factor 2 (FGF2). Next, the cells were differentiated into small intestinal stem-like cells by culturing them in advanced DMEM/F-12 containing 2% FBS, 1% GlutaMax, 1% penicillin-streptomycin, and FGF2. Small intestinal stem-cell-like cells were dissociated and seeded on cell culture inserts coated with GFR-Matrigel. After seeding, the cells were cultured in advanced DMEM/F-12 containing EGF and forskolin, 2% FBS, 1% nonessential amino acids (NEAA), 2 mM L-Glu, 1% penicillin-streptomycin, 2% B27 supplement, 1% N2 supplement, and 1% HepExtend supplement. Then, PD98059, 5-aza-2-dC, and A-83-01 were added to induce the cells to differentiate into small intestinal epithelial cells. The culture medium was changed every 2 days during the preculture.

### 2.4. Cell Culture Using the KIM-Plate

PXB cells were monocultured in the KIM-Plate to investigate the effect of the perfusion culture. PXB cells precultured on the cell desk were introduced into chamber A of the KIM-Plate. After three days of perfusion culture in the KIM-Plate, the morphology of the cells was observed. Static culture of PXB cells on the KIM-Plate was performed as a control experiment for the perfusion culture.

**Figure 2:**
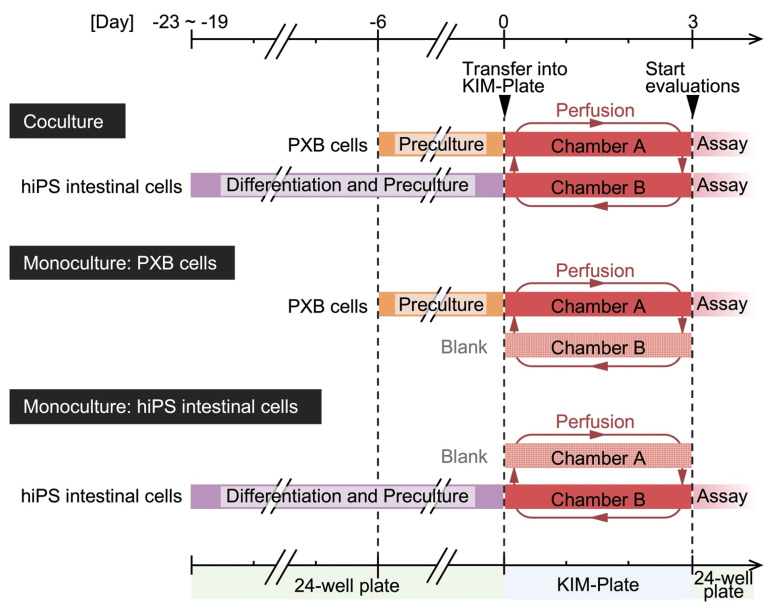
Experimental schedule for coculture and monoculture using the kinetic pump integrated microfluidic plate (KIM-Plate). Cells were seeded onto cell culture inserts or cell desks. Cell differentiation, preculture, and functional evaluation were performed in conventional 24-well plates, and cells on the culture inserts and cell desks were introduced into the KIM-Plate only during perfusion culture.

hiPS intestinal cells and PXB cells were cocultured as gut and liver model cells in the KIM-Plate to evaluate the plate’s usefulness as an evaluation system for interorgan interactions in vitro. PXB cells precultured on the cell desk and hiPS intestinal cells precultured on the culture insert were respectively introduced into chambers A and B of the KIM-Plate on day 0 (Figure 2). The transepithelial electrical resistance (TEER) values of hiPS intestinal cells were measured on day 3 using Millicell-ERS (MERS00002, Merck KGaA, Darmstadt, Germany). To evaluate the effect of coculture on hiPS intestinal cells and PXB cells individually, the cells on the cell desk or the culture insert were transferred to 24-well plates. Then, TEER, function activity, and gene expression levels of the cells were measured. Monocultures of hiPS intestinal cells and PXB cells were performed as a control experiment for coculture.

The rotation speed of the kinetic pump was set to 6500 rpm for the cell culture experiments using the KIM-Plate. The preculture medium for PXB cells with 2% FBS was used as the medium for the perfusion culture in the KIM-Plate. A total of 2000 µL of the culture medium was introduced into each MO-MPS unit, and an additional 240 µL was introduced into the apical side of the culture insert. 

### 2.5. Quantitative Reverse Transcription Polymerase Chain Reaction (qRT-PCR) 

RNA from hiPS intestinal cells and PXB cells in monoculture and coculture was extracted using QIAzol (79306, QIAGEN, Hilden, Germany) and TRIzol (15596-018, Invitrogen, Waltham, MA, USA), respectively. RNA was purified from hiPS intestinal cells and PXB cells using the RNeasy Plus Mini kit (74134, QIAGEN, Hilden, Germany) and a Direct-zol RNA Microprep (R2062, Zymo Research, Irvine, CA, USA), respectively, according to the manufacturer’s instructions. Purified RNA samples were qualitatively and quantitatively analyzed using the NanoDrop Lite spectrophotometer (Thermo Fisher Scientific, Waltham, MA, USA). RNA (500 ng from each sample) purified from hiPS intestinal cells and PXB cells was reverse transcribed using the High-Capacity RNA-to-cDNA kit (4388950, Applied Biosystems, Waltham, MA, USA) and iScript cDNA Synthesis kit (1708891, Bio-Rad Laboratories Inc., Hercules, CA, USA), respectively, following the manufacturer’s protocol. The gene expression levels of the transcribed complementary DNA samples from hiPS intestinal cells and PXB cells were evaluated using a Taqman probe and the TaqMan Gene Expression Master Mix (4369016, Applied Biosystems, Waltham, MA, USA) and SSo Advanced SYBR Green Supermix (172-5271, Bio-Rad Laboratories, Inc., Hercules, CA, USA), respectively, and detected using the CFX Connect Real-Time PCR detection system (Bio-Rad Laboratories, Inc., Hercules, CA, USA) and gene-specific primers (Tables S1 and S2).

### 2.6. Cell Activity Evaluation Using High-Performance Liquid Chromatography-Tandem Mass Spectrometry (LC-MS/MS)

The transporter and metabolic activities of cells were evaluated using each substrate mixture. The transporter activity of hiPS intestinal cells was evaluated using an efflux substrate mixture containing 1 µM quinidine for P-glycoprotein (P-gp). The efflux substrate mixture was introduced into either the AP or BL side to evaluate the polar transport of the substance, and HBSS (+) (084-08965, FUJIFILM Wako Pure Chemical Co., Osaka, Japan) was introduced into the other side. A metabolic substrate mixture containing 1 µM diclofenac, 1 µM midazolam, and 10 µM irinotecan for CYP2C9, CYP3A4, and CES2, respectively, was introduced on both sides of the culture inserts to evaluate the metabolic activity of hiPS intestinal cells. The metabolic activity of the PXB cells was determined using a CYP substrate mixture containing 20 µM phenacetin, 2 µM coumarin, 5 µM bupropion, 0.1 µM amodiaquine, 1 µM diclofenac, 40 µM (S)-mephenytoin, 5 µM bufuralol, and 2 µM midazolam for CYP1A2, CYP2A6, CYP2B6, CYP2C8, CYP2C9, CYP2C19, CYP2D6, and CYP3A4, respectively. hiPS intestinal cells were incubated with HBSS (+) for 60 min and then exposed to the efflux substrate mixture for 60 min for transporter activity evaluation. hiPS intestinal cells and PXB cells were exposed to each substrate mixture for 60 min to evaluate their metabolic functions. For the activity evaluation of hiPS intestinal cells, 150 µL was introduced into the AP side and 600 µL was introduced into the BL side, whereas for the activity evaluation of PXB cells, 1000 µL of the substrate mixture was introduced into the wells. All substrate mixtures were prepared with HBSS (+). The cells were washed once with HBSS (+) and then exposed to the substrate mixture at 37 °C in an incubator with humidified atmosphere. The concentrations of substrates and metabolites in each sample collected from 0 to 60 min were measured using LC-MS/MS.

The collected sample (20 µL) was mixed with 150 µL of methanol and 30 µL of water containing 1 µM of imipramine as an internal standard. After vortexing, the solutions were centrifuged at 15,000 rpm for 10 min at 4 °C, and the resulting supernatants were injected into the LC-MS/MS system. The amount of each metabolite was determined using an LCMS8050 triple quadrupole mass spectrometer (SHIMADZU CORPORATION, Kyoto, Japan) coupled with an LC-30A system (SHIMADZU CORPORATION, Kyoto, Japan). Chromatography was performed using a CAPCELL PAK C18 MG III column (ID 2.0 × 50 mm; Osaka Soda Co., Ltd., Osaka, Japan) at 50 °C through step-gradient elution with a flow rate of 0.4 mL/min according to the following program: 0–0.6 min, 95% A/5% B; 0.6–3.0 min, 95% A/5% B to 10% A/90% B; 3.0–4.0 min, 10% A/90% B; 4.0–4.1 min, 10% A/90% B to 95% A/5% B; 4.1–5.5 min, 95% A/5% B; (A, water containing 0.1% formic acid; B, acetonitrile containing 0.1% formic acid). The detected mass numbers and collision energy (CE) are shown in Table S3.

### 2.7. Statistical Analysis

All values are expressed as the mean ± SD of experiments performed at least in triplicates. Statistical analysis was performed using the Student’s *t*-test, and differences were considered statistically significant at a *p* value of <0.05. The Smirnoff-Grubbs test was performed, and all data outliers were excluded. 

## 3. Results and Discussion

### 3.1. Evaluation of the Stirrer-Based Kinetic Pump

**Figure 3:**
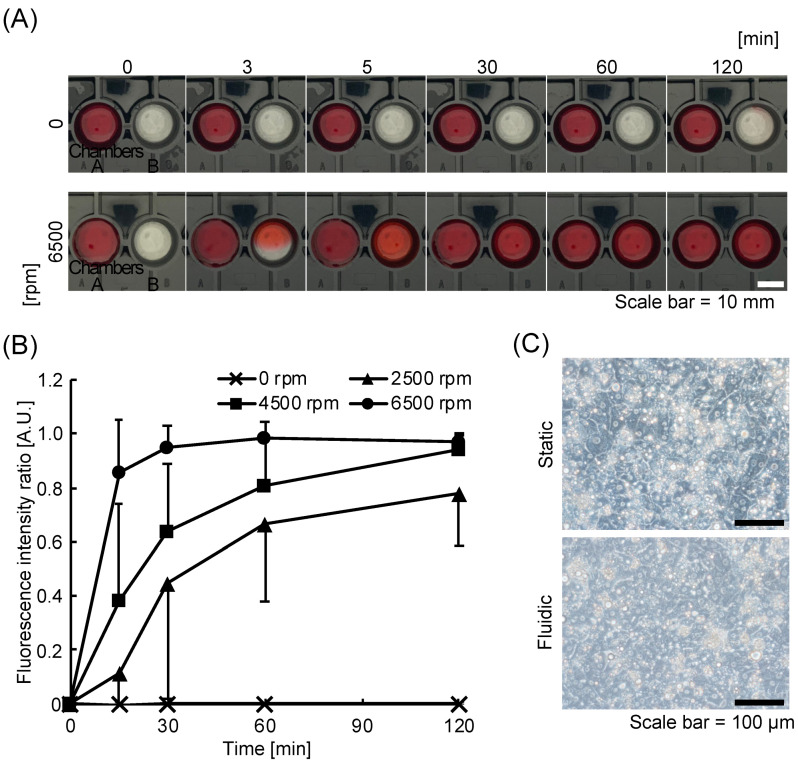
Evaluation of a stirrer-based kinetic pump on the kinetic pump integrated microfluidic plate (KIM-plate). (**A**) Comparison of chemical transportation with/without stirrer bar rotation of the kinetic pump in the KIM-plate. The red dye diffused from chamber A to chamber B by the rotation of the kinetic pump. (**B**) Time-dependent change in the fluorescence intensity ratio (chamber B/chamber A) of Lucifer yellow applied into chamber A. Transportation was accelerated by increasing the rotation speed of the stirrer bar. Each datum represents the mean ± SD (*n* = 7–13). (**C**) Photographs of PXB cells in static/fluidic conditions. No difference in PXB cell number or morphology was observed between the nonperfusion and perfusion cultures.

The transportation experiment using the red dye solution was conducted to evaluate the function of the kinetic pump on the KIM-Plate. Transportation was not observed at 0 rpm, while the red dye was transported into chamber B in about 5 min at 6500 rpm (Figure 3A), indicating that the kinetic pump perfused the solution and facilitated the transportation of the substance. The transportation evaluation experiment using LY for quantitative evaluation also showed that the kinetic pump promoted the transportation of the substance (Figure 3B). The fluorescence intensity ratio (chamber B/chamber A) at 120 min was the same as that at 0 min when the rotation speed was 0 rpm. The fluorescence intensity ratio increased with time at 2500–6500 rpm, and the rate of increase in the fluorescence intensity ratio also improved with the increase in the rotation speed of the kinetic pump. The concentration of LY in chambers B and A became uniform in approximately 30 min at 6500 rpm and 120 min at 4500 rpm. The flow rate increased with the rotation speed of the kinetic pump in the KIM-Plate, which has open-type culture chambers (Movie S1 and Figure S2), as well as in the closed-type MO-MPS that we previously developed [33,35,36]. This indicates that the kinetic pump-induced circulation in the KIM-Plate promotes substance transportation between chambers and that the transportation speed can be controlled by the rotation speed of the kinetic pump. 

PXB cells were cultured in the KIM-Plate to evaluate the effect of perfusion culture. The number and morphology of PXB cells in perfusion and static culture did not differ on day 3 of culture (Figure 3C). This result indicates that there is no effect on the number and morphology of the cells under perfusion condition in the KIM-Plate.

### 3.2. The KIM-Plate Enabled Coculture of hiPS Intestinal Cells and Primary Hepatocytes

**Figure 4:**
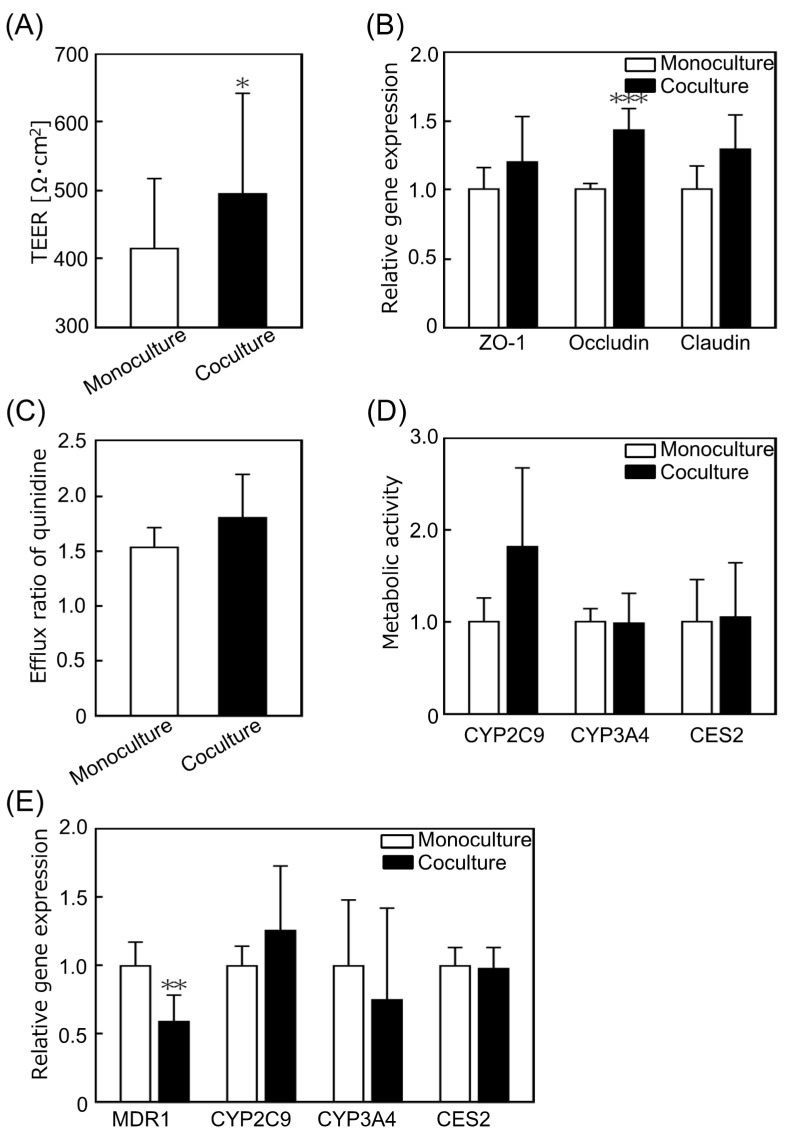
Evaluation of the effects of coculture on the function of human induced pluripotent stem cell derived small intestinal epithelial cells (hiPS intestinal cells). (**A**) Transepithelial electrical resistance (TEER) value of hiPS intestinal cells on day 3. The TEER value was significantly increased by coculture (*n* = 23–26). (**B**) The relative gene expression levels associated with tight junctions in hiPS intestinal cells. The gene expression levels tended to increase with coculture (*n* = 5–6). The gene expression levels were normalized to those of the monoculture. (**C**) Efflux ratio (ER) of quinidine, a P-glycoprotein (P-gp) substrate. The ER values were not significantly different between the coculture and monoculture (*n* = 3). (**D**) Metabolic activities of CYP2C9, CYP3A4, and CES2 in hiPS intestinal cells. The metabolic activities were not significantly different between the coculture and monoculture. Each value was normalized to the enzyme metabolic activities of the monoculture (*n* = 6). (**E**) The relative gene expression levels in hiPS intestinal cells. The expression level of MDR1 was significantly decreased by coculture, and there were no significant differences in the expression levels of other genes (*n* = 5–6). Each value was normalized to that in the monoculture for each gene. Data represent the mean ± SD, * *p* < 0.05, ** *p* < 0.01, and *** *p* < 0.005.

The tight junction function of hiPS intestinal cells was evaluated from multiple perspectives to determine the effect of coculture of small intestinal cells and hepatocytes on the tight junctions of hiPS intestinal cells. The TEER value of cocultured hiPS intestinal cells using the KIM-Plate for 3 days was significantly higher than that of the monoculture (Figure 4A). Gene expression levels related to tight junction formation were measured using qRT-PCR. The expression level of zonula occludens-1 (ZO-1) showed no significant difference, while that of occludin was significantly increased, and that of claudin (*p* = 0.068) also showed a tendency to increase following coculture compared to the monoculture expression levels (Figure 4B). These results suggest that the tight junctions of hiPS intestinal cells become stronger following coculture with PXB cells. Zeller et al. reported that coculturing with hepatocytes reduced the permeability of Sertoli cells via junction protein relocation to cell membrane and/or increase in protein quantity [38]. In this study, it is considered that the TEER value was increased because the number of junction proteins on the cell membrane increased with the increase in the gene expression levels of occludin and claudin in hiPS intestinal cells following coculture with PXB cells. 

The transporter and metabolic functions of hiPS intestinal cells cocultured with hepatocytes were evaluated from various aspects using LC-MS/MS and qRT-PCR. The apparent drug permeability coefficient (Papp, in units of centimeters per minute) was calculated according to the following equation: Papp = dQ/dt/(A × C_0_), where Q, A, and C_0_ are the amount of substrate transported over time, the area of the membrane surface, and the initial concentration of substrate in the donor medium, respectively. The efflux ratio (ER), which indicates the transporter activity, was calculated by dividing the Papp B to A by Papp A to B, obtained based on the results of LC-MS/MS analysis. The ER of quinidine, a P-gp substrate involved in drug excretion, showed no significant difference between the monocultures and cocultures (Figure 4C). Metabolic activity was evaluated as the amount of metabolite per drug exposure calculated by dividing the amount of metabolite at 60 min by the area under the curve (AUC) of the substrate compound. The results of metabolic activity evaluation showed that CYP2C9 (*p* = 0.093) metabolic activity trended to increase with coculture, while the metabolic activities of CYP3A4 and CES2 were not significantly different from those of the monoculture (Figure 4D). The gene expression levels related to drug excretion (MDR1) and metabolism (CYP2C9, CYP3A4, and CES2) of hiPS intestinal cells were examined using qRT-PCR. The expression level of MDR1, related to P-gp, was significantly decreased by coculture, while those of other genes did not show significant differences (Figure 4E). In this experiment, there was little change in the metabolic function of hiPS intestinal cells as a result of coculture with hepatocytes. These results are similar to those of our previous report of coculture of PXB cells with hiPS-derived small intestinal cells [39]. Kabeya et al. reported that the ER of P-gp was 4.2 and that of cells exposed to inhibitors was 0.8 in hiPS cell-derived small intestinal epithelial cells [37]. The ER of P-gp obtained in this experiment was similar to that of cells exposed to inhibitors in a previous study, indicating that the drug excretion function of hiPS intestinal cells was reduced. The culture medium used in this study was optimized for PXB cells and lacked the compounds necessary to maintain their function as intestinal model cells. Therefore, the function of hiPS intestinal cells was probably greatly impaired by the medium, and the coculture effect was, therefore, hardly observed.

**Figure 5:**
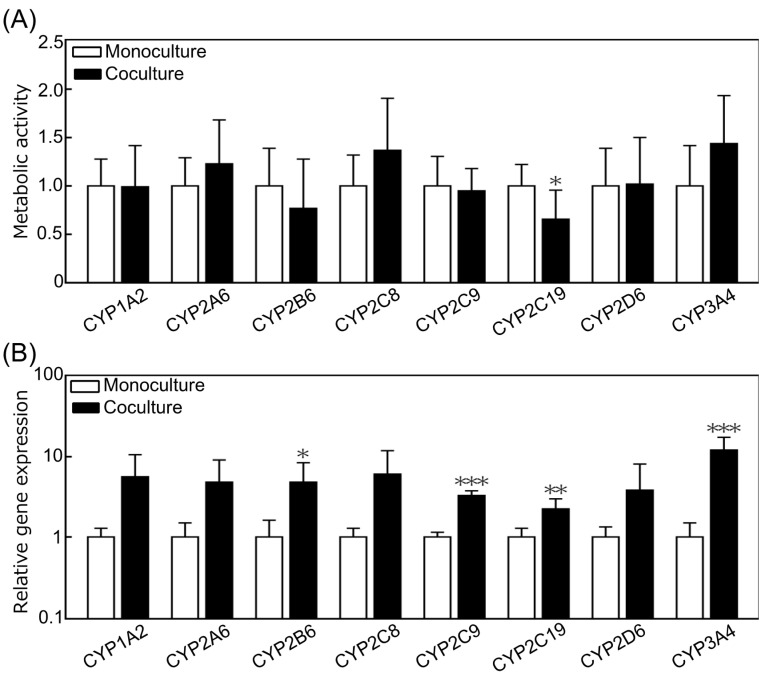
Evaluation of the coculture effects on the metabolic function of primary hepatocytes derived from chimeric mice with humanized liver tissues (PXB cells). (**A**) Metabolic activity of PXB cells normalized by monoculture using liquid chromatography–tandem mass spectrometry (LC-MS/MS). The metabolic activity of CYP2C19 was significantly decreased by coculture (*n* = 5–8). (**B**) The relative gene expression levels related to the metabolism of PXB cells tended to increase in coculture. Significant differences were observed in the expression levels of CYP2B6, 2C9, 2C19, and 3A4 (*n* = 7–18). Each value was normalized to that of the monoculture for each gene. Data represent the mean ± SD, * *p* < 0.05, ** *p* < 0.01, and *** *p* < 0.005.

The metabolic activities and gene expression levels of CYP family members (1A2, 2A6, 2B6, 2C8, 2C9, 2C19, 2D6, 3A4), representative metabolic enzymes in hepatocytes, were investigated to evaluate the effect of coculture with small intestinal cells on PXB cells. In coculture, the metabolic activity of CYP2C19 was significantly decreased, and that of CYP3A4 (*p* = 0.0599) showed an increasing trend, but the metabolic activity of other enzymes showed no difference compared to that of the monoculture (Figure 5A). In coculture, the gene expression levels of CYP2B6, 2C9, 2C19, and 3A4 were significantly increased, and those of CYP1A2 (*p* = 0.0569), CYP2A6 (*p* = 0.0726), and CYP2C8 (*p* = 0.0833) tended to increase (Figure 5B). Despite the increase in gene expression of major metabolic enzymes, the results of drug metabolism assays did not show any improvement in activity. The reason for this result can be attributed to the complexity of drug metabolism. Multiple metabolites are produced for a substrate compound because drug metabolism involves a complex interplay of phase I metabolic processes, such as oxidation, reduction, and hydrolysis, followed by phase II metabolic processes, such as glucuronidation and glutathione conjugation. For example, (S)-mephenytoin, which is a substrate for CYP2C19, produces (S)-4′-hydroxymephenytoin (4OHM) during CYP2C19 metabolism and (S)-N-demethyl metabolite during CYP2B6 or 2C9 metabolism, and 4OHM undergoes metabolic conjugation by UDP-glucuronosyltransferase (UGT) [40]. Moreover, it is widely known that increased metabolic enzyme mRNA expression does not always correlate with increased metabolic functions. For these reasons, a significant difference in metabolic activity between cocultures and monocultures might not be observed. However, the trend of increased mRNA expression found in our previous studies using a different MPS was also observed in this study [39]. Hence, we can conclude that the KIM-Plate is sufficiently functional, reliable, and useful as a coculture experimental system. 

The gene expression level of PXB cells increased with the coculture in this study, but the effect of coculture needs to be further verified in future studies. The development of an optimized culture medium to maintain multiple cells is essential to verify the coculture effects. It is necessary to use a culture medium optimized for each cell type to maintain the function of primary cultured cells and iPS-derived cells, as shown in this experiment where the function of hiPS intestinal cells was reduced by the PXB-cell medium. If the function of one or both cell types is reduced during coculture, as the results of this experiment showed, the coculture effect may not be fully evaluated. In the future, with the maturation of MPS technology and establishment of coculture experimental systems, the development of a standard culture medium that can simultaneously maintain the functions of many cell types will be required. The standard culture medium is more important for MPSs, such as the KIM-Plate, which can be used to establish and handle organ models with advanced functions.

Most conventional MPSs require an external pump system, such as syringe pumps, peristaltic pumps, and air pressure controllers, to perfuse culture medium through multiple cell culture chambers, and they should be connected to each other with a lot of tubes. This causes complicated handling, which reduces the throughput of experiments [1,17,27]. The KIM-Plate with on-chip pumping functions with a simple structure is simple to operate and does not compromise the throughput of experiments like conventional multiwell plates. The KIM-Plate is only put on the stirrer motor bases for the perfusion of culture medium. No tubing is required. In the future, the KIM-Plate culture chamber can be changed from 24-well plate size to 48-well or 96-well size to further increase the throughput. However, in order to increase the number of culture chambers, it is necessary to further reduce the size of the kinetic pumps and the stirrer motors. It may be necessary to develop a kinetic pump with better pumping efficiency. Solving the technical issues related to the kinetic pump will further increase the throughput of experiments using the KIM-Plate. 

It well known that shear stress to cells, such as endothelial and kidney cells, maintains and induces their original functions [1,5]. The kinetic pump proposed in this study does not generate shear stress onto cells due to the structure of the cell culture chambers and the microchannels. If cells that require shear stress are used, it may be possible to modify the structure or use a jig to generate shear stress onto cells in the culture chambers. Although the KIM-Plate still has some room for improvement, it is expected to bring many benefits to cell-based assays in the fields of drug discovery and biology.

## 4. Conclusions

In this study, we developed a stirrer-based kinetic pump integrated microfluidic plate as a novel MO-MPS platform for easy evaluation of interorgan/tissue-tissue interactions in cell-based assays. The KIM-Plate is a simple structure consisting of 24-well sized culture chambers connected by microchannels, allowing the use of conventional culture inserts and culture methods. The perfusion function of the kinetic pump in the KIM-Plate was evaluated using transportation evaluation experiments, which showed that the KIM-Plate had sufficient agitation effect. Coculture experiments with PXB cells and hiPS intestinal cells showed that coculture of normal cells increased the gene expression levels related to the metabolism of PXB cells and the TEER of hiPS intestinal cells. We confirmed that the KIM-Plate can be used to evaluate the effect of coculture on each cell type using conventional cell-based assay methods, such as LC-MS/MS and qRT-PCR. These results indicate that the KIM-Plate is useful for cell-based assays. In addition, cell differentiation, preculture, and evaluation can be performed on conventional 24-well plates by using culture inserts or cell desks in the open-type culture chambers of the KIM-Plate. Using this system, cell-based assays can be performed by installing culture inserts or cell desks into the KIM-Plate only during coculture. The greatest advantage of the KIM-Plate proposed in this study is that it can be used for coculture in the same way as conventional culture dishes. Conventional MPSs are complicated to set up and handle, and their throughput is quite low. The KIM-Plate, which realizes on-chip pumping functions with a simple structure, is expected to bring many advantages to cell-based assays in the fields of drug discovery and biology because of its high throughput.

## Supplementary Materials

The following are available online at https://www.mdpi.com/article/10.3390/mi12091007/s1, Table S1: Primer sequences used for qRT-PCR of PXB cells, Table S2: Assay ID of the probe used for qRT-PCR of hiPS intestinal cells, Table S3: The substrate mixture used for activity evaluations, Table S4: The compounds used in the adsorption experiments, Figure S1: Graph of the amount of compound adsorbed in the KIM-Plate vs. conventional 24-well plate, Movie S1: Flow visualization movie of fluorescent beads in the KIM-Plate, Figure S2: Flow rate on the KIM-Plate.
