# Supplementary material for: A Kinetic Pump Integrated Microfluidic Plate (KIM-Plate) with High Usability for Cell Culture-Based Multiorgan Microphysiological Systems
Source: Micromachines (Basel). 2021 Aug 24;12(9):1007. doi: 10.3390/mi12091007 (PMC8471412; doi:10.3390/mi12091007)
Supplement: Supplementary file 1 [file micromachines-12-01007-s001.zip › micromachines-1329030-supplementary.pdf]

Supplementary Materials: A Kinetic Pump Integrated Microfluidic Plate (KIM-Plate) with High Usability for Cell Culture-Based Multiorgan Microphysiological Systems

Kenta Shinha, Wataru Nihei, Hiroko Nakamura, Tomomi Goto, Takumi Kawanishi, Naoki Ishida, Nao Yamazaki, Yuki Imakura, Shinji Mima, Kosuke Inamura, Hiroshi Arakawa, Masaki Nishikawa, Yukio Kato, Yasuyuki Sakai, Hiroshi Kimura

Table S1. Primer sequences used for qRT-PCR of primary hepatocytes derived from chimeric mice with humanized liver tissues (PXB-cells).

Target	Forward	Reverse
GAPDH	TGAAGACGGGCGGAGAGAAA	CCAATACGACCAAATCCGTTGAC
CYP1A2	CAGCTTCCTCATCCTCCTGCTA	AGGCTGAGCATCTCATCGCTAC
CYP2A6	CCGTGTTCACCATTCACTTG	TTGCTGAATACCACGCCATA
CYP2B6	CTGCCTCTGAGAGACCTGCT	GCAGGAGAGTTGCTTGATCC
CYP2C8	GAACACCAAGCATCACTGGA	AGCAGGAGCAGGAGTCCATA
CYP2C9	GGACAGAGACGACAAGCACA	CATCTGTGTAGGGCATGTGG
CYP2C19	GAACACCAAGAATCGATGGACA	TCAGCAGGAGAAGGAGAGCATA
CYP2D6	CAGAGATGGAGAAGGCCAAG	CCCTATCACGTCGTCGATCT
CYP3A4	ACATAGCCCAGCAAAGAGCAAC	GTCTGGGATGAGAGCCATCACT

Table S2. Assay ID of the probe used for qRT-PCR of human induced pluripotent stem cell-derived small intestinal epithelial cells (hiPS-intestinal cells).

Gene	Assay ID
GAPDH	Hs02758991_g1
TJP1	Hs01551867_m1
OCLN	Hs00170162_m1
CLDN1	Hs00221623_m1
ABCB1	Hs00184500_m1
CYP2C9	Hs02383631_s1
CYP3A4	Hs00604506_m1
CES2	Hs01077945_m1

Table S3. Substrates used for activity evaluations.

Mixture name	Substrate	μM	Target	detected mass numbers	collision energy (CE)	Metabolite	detected mass numbers	collision energy (CE)
Efflux mixture	Quinidine	1	P-gp	325.2 > 160.1	− 28 V	-----	-----	-----
Metabolic mixture	Diclofenac	1	CYP2C9	294.1 > 214.3	− 21 V	4'-OH diclofenac	312.0 > 230.0	− 32 V
	Midazolam	1	CYP3A4	326.0 > 209.0	− 35 V	1-OH Midazolam	342.0 > 203.0	− 27 V
	Irinotecan	10	CES2	587.4 > 124.2	− 35 V	SN-38	393.3 > 349.2	− 26 V
CYP mixture	Phenacetin	20	CYP1A2	180.25 > 110.2	− 22 V	Acetaminophen	152.0 > 110.0	− 9 V
	Coumarin	2	CYP2A6	147.1 > 103.1	− 18 V	7-OH coumarin	163.0 > 107.0	− 24 V
	Bupropion	5	CYP2B6	240.3 > 131.2	− 26 V	OH bupropion	256.0 > 238.0	− 13 V
	Amodiaquine	0.1	CYP2C8	356.3 > 283.2	− 21 V	N-Desethylamodiaquine	328.0 > 283.0	− 18 V
	Diclofenac	1	CYP2C9	294.1 > 214.3	− 21 V	4'-OH diclofenac	312.0 > 230.0	− 32 V
	(S)-Mephenytoin	40	CYP2C19	217.2 > 188.1	− 16 V	4'-OH mephenytoin	235.1 > 150.1	− 17 V
	Bufuralol	5	CYP2D6	262.3 > 188.25	− 17 V	1'-OH bufuralol	278.0 > 186.0	− 19 V
	Midazolam	2	CYP3A4	326.0 > 209.0	− 35 V	1'-OH midazolam	342.0 > 203.0	− 27 V
internal standard	imipramine		transporter study	281.0 > 86.0	− 17 V	-----	-----	-----
	niflumic acid		metabolic study	283.25 > 265.2	− 21 V	-----	-----	-----

Experiment S1: Substance adsorption test

We evaluated the adsorption of several compounds on the kinetic-pump integrated microfluidic plate (KIM-Plate). A total of 2 mL of a compound mixture containing 1 μM quinidine, 10 μM sulfasalazine, 10 μM rosuvastatin, 1 μM propranolol, 1 μM diclofenac, 2 μM midazolam, 10 μM irinotecan, 2 μM coumarin, 1 μM 7-OH coumarin, 20 μM phenacetin, 5 μM bupropion, 0.1 μM amodiaquine, 40 μM (S)-mephenytoin and 5 μM bufuralol (Table S4), was introduced into the KIM-Plate and perfused by the stirrer-based kinetic-pump at 6,500 rpm for 24 h. The concentration of the compounds in the samples collected after 24 h was measured using liquid chromatography tandem mass spectrometry (LC-MS/MS). The concentration of the compounds in the samples after 24 h weas comparable between the 24-well plates and the KIM-Plate (Figure S1). Polydimethylsiloxane (PDMS), which is widely used as a material for MPS, is well known to adsorb substances. These indicate that the adsorption of drug compounds on the KIM-Plate is comparable to that of conventional culture dishes and that drug adsorption on the KIM-Plate does not need to be considered as much as that of microphysiological systems (MPSs) made of PDMS during cell-based assays.

Table S4. Concentrations of the compounds used in the adsorption experiments and liquid chromatography tandem mass spectrometry (LC-MS/MS) information.

Substrate	μM	detected mass numbers	collision energy (CE)
Quinidine	1	325.2 > 160.1	− 28 V
Sulfasalazine	10	397.1 > 197.3	− 22 V
Rosuvastatin	10	482.0 > 258.2	− 34 V
Propranolol	1	260.2 > 116.2	− 17 V
Diclofenac	1	294.1 > 214.3	− 21 V
Midazolam	2	326.0 > 209.0	− 35 V
Irinotecan	10	587.4 > 124.2	− 35 V
7-OH coumarin	1	163.0 > 107.0	− 24 V
Phenacetin	20	180.25 > 110.2	− 22 V
Coumarin	2	147.1 > 103.1	− 18 V
Bupropion	5	240.3 > 131.2	− 26 V
Amodiaquine	0.1	356.3 > 283.2	− 21 V
(S)-Mephenytoin	40	217.2 > 188.1	− 16 V
Bufuralol	5	262.3 > 188.25	− 17 V

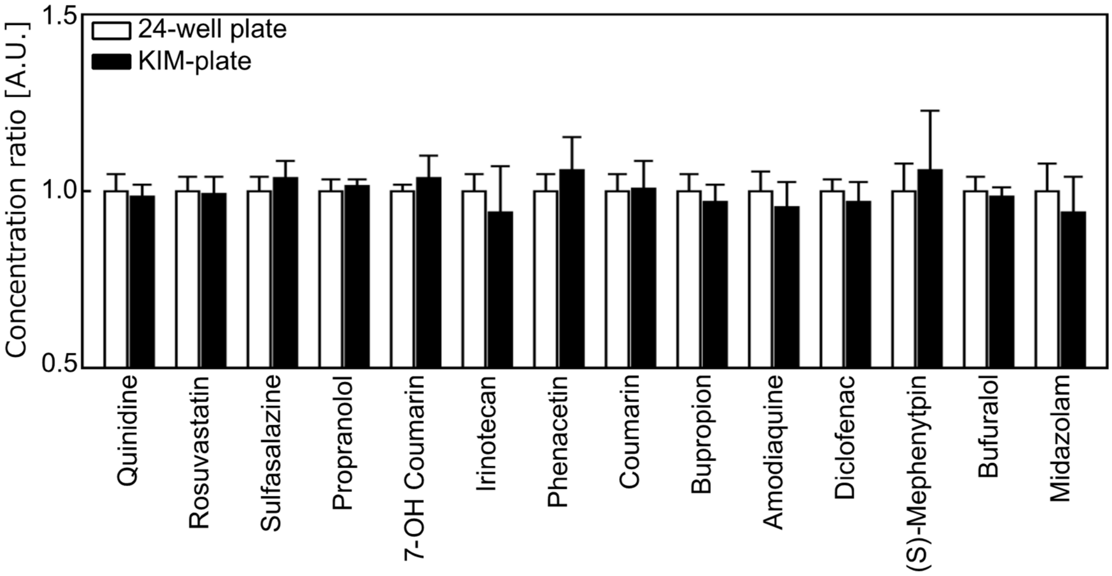

Figure S1. The concentration was normalized to that of the 24-well plate for each compound. The amount of compound adsorbed on the kinetic-pump integrated microfluidic plate (KIM-Plate) was similar to that on the 24-well plate (n=6). Data represent the mean ± SD.

Experiment S2. Flow visualization in the multi-organ microphysiological system (MO-MPS) unit

The flow in the kinetic-pump integrated microfluidic plate (KIM-Plate) was visualized using fluorescent microbeads with a diameter of 1.17 μm (17687, Polysciences Inc., Warrington, PA, USA) to evaluate the function of the kinetic-pump. To observe the inside of microchannels, a mock-up model of the MO-MPS unit was fabricated with transparent polymethyl methacrylate and used for the experiment. The fluorescent microbeads were suspended in ultra-pure water with 0.1% Tween20 (103168, MP Biomedicals, Irvine, CA USA). The suspensions were introduced into the culture chambers and the stirrer-based kinetic-pump was driven at 900-4,500 rpm of the stirrer bar. The flowing microbeads in the microchannels were observed using a fluorescence microscope (IX71, Olympus Corporation, Tokyo, Japan) (Movie S1). The flow rates were calculated from the velocities of the flowing fluorescent bead in the movies measured using ImageJ (NIH). The flow rate increased with the rotation speed of the stirrer bar of the kinetic-pump (Figure S2). This result shows that the flow rate is controllable by controlling the rotation speed of the stirrer bar. This trend was similar to that of the closed type MO-MPS we previously developed [33,35,36]. Therefore, we conclude that the kinetic-pump is an effective pumping method for the KIM-Plate with open type culture chambers.

Movie S1. Flow visualization movie of fluorescent beads in the kinetic-pump integrated microfluidic plate (KIM-Plate). The flow velocity increased with the increase in the rotation speed of the kinetic-pump. A bar is 500 μm.

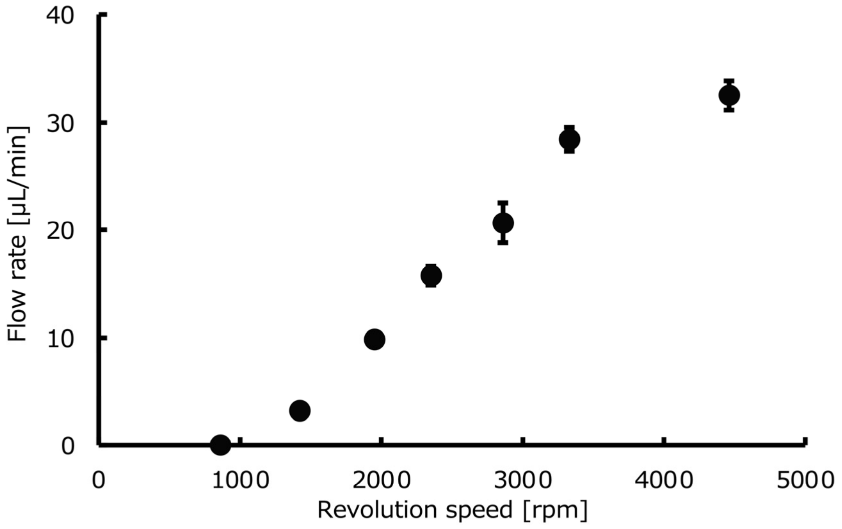

Figure S2. Flow rate on the KIM-Plate at 900–4,500 rpm of the rotation speed of the stirrer bar. The flow rate increased with the rotation speed of the stirrer bar of the kinetic-pump. Data represents the mean ± SD (n=3).
